# Agriculture increases the bioavailability of silicon, a beneficial element for crop, in temperate soils

**DOI:** 10.1038/s41598-020-77059-1

**Published:** 2020-11-17

**Authors:** M. Caubet, S. Cornu, N. P. A. Saby, J.-D. Meunier

**Affiliations:** 1 INRAE, Infosol, US 1106, Orléans, France; 2 Aix-Marseille Univ, CNRS, IRD, Coll de France, INRAE, CEREGE, Aix-en-Provence, France

**Keywords:** Biogeochemistry, Environmental sciences

## Abstract

Crops may take benefits from silicon (Si) uptake in soil. Plant available Si (PAS) can be affected by natural weathering processes or by anthropogenic forces such as agriculture. The soil parameters that control the pool of PAS are still poorly documented, particularly in temperate climates. In this study, we documented PAS in France, based on statistical analysis of Si extracted by CaCl_2_ (Si_CaCl2_) and topsoil characteristics from an extensive dataset. We showed that cultivation increased Si_CaCl2_ for soils developed on sediments, that cover 73% of France. This increase is due to liming for non-carbonated soils on sediments that are slightly acidic to acidic when non-cultivated. The analysis performed on non-cultivated soils confirmed that Si_CaCl2_ increased with the < 2 µm fraction and pH but only for soils with a < 2 µm fraction ranging from 50 to 325 g kg^−1^. This increase may be explained by the < 2 µm fraction mineralogy, i.e. nature of the clay minerals and iron oxide content. Finally, we suggest that 4% of French soils used for wheat cultivation could be deficient in Si_CaCl2_.

## Introduction

Silicon (Si) has been shown to be beneficial for crops exposed to biotic or abiotic stresses^1–5^. The role of Si remains controversial^1,6^, but crop yields may depend on the bioavailable Si in the soil^7^, particularly for globally cultivated crops and staple food that contain 1% or more Si by dry weight such as rice, sugar cane, or wheat, or more broadly, the Poaceae^8–10^. Soil Si bioavailability is therefore an emerging issue in agriculture, as Si fertilization of soil depleted of bioavailable Si could increase yields^8,11–13^.

Si is taken up from the soil solution by roots and is accumulated in the shoots in the form of amorphous silica particles (phytoliths). In the soil solution, dissolved Si (DSi) primarily occurs in the neutral form Si(OH)_4_ (silicic acid). The DSi concentration typically ranges from 0.1 to 0.6 mmol L^−1^^3^. DSi originates from the dissolution of primary and secondary silicate minerals through alteration or chemical weathering^14^ and from plant recycling through the dissolution of phytoliths^15,16^. The alterability of silicate minerals can be characterized by their solubility and kinetic properties, which are dependent upon temperature, pH, and available solutes^17,18^.

DSi concentration in the soil solution can be lowered by plant uptake^19^, uptake by silica-shelled microorganisms^20^ and Si adsorption to mineral surfaces^21^. Quantifying the pool of Si that is bioavailable (PAS) is still a challenge^7^. Si extracted with CaCl_2_ (Si_CaCl2_), acetate, acetic acid, or citrate are used as PAS proxies since the concentrations extracted by these reagents are proportional to plant Si concentration or grain yield^22,23^. PAS concentrations extracted by these reagents have been shown to be positively correlated with soil properties such as phytolith content^24^, pH^25–27^, < 2 µm fraction^7,28^, organic matter, and iron oxides^28^. A negative correlation between PAS and total Si content has also been documented, reflecting the predominance of low solubility minerals such as quartz in the Si pool^28,29^. Critical levels of PAS have been identified in cultivated lands mainly under tropical climate including the paddy soils of Asia^7,30^, the USA^31^, and Brazil^32^, and the sugarcane fields of Australia^33^. Under critical levels of PAS, silicate fertilization may be recommended and is already applied on tropical soils in some countries.

Chemical weathering leads to a removal of Si through leaching and erosion^14^. Intense weathering causes soils to become acidic and depleted in bases and Si (desilication) due to loss of primary silicates that are easily weatherable. Cultivation, through exportation of crops containing silicon has been indicated as a Si pool modifier by lowering the phytolith pool^34–39^; as a consequence, cultivation may reduce the soil Si availability if plant residues are not returned to the fields^40^. Darmawan et al.^11^ documented a significant decrease of available Si after approximately 30 years of intensive rice cultivation in Indonesia. The decrease of PAS in soil has been listed as a factor contributing to the stagnation of crop yields^12^. Si supply is considered a useful strategy for improving crop health^7^, the influence of land use and agricultural practices on PAS has become an emerging issue^41^.

The objectives of this paper are to determine the impact of agriculture on PAS in temperate soils. To do so we (i) estimated the spatial variability of PAS in France, a country with a high soil diversity^42^ that can be considered as representative of most European soils, and likely be of most temperate soils worldwide; (ii) hierarchized the soil characteristics governing this variability in non-cultivated soils for different soil groups (pH, < 2 µm fraction, cation exchange capacity (CEC), major elements) to (iii) understand how and under which pedological conditions, cultivation acts on these soil divers; and (iv) determine which soils may exhibit too low Si_CaCl2_ concentrations in soil solution for proper growth of wheat, a Si accumulator plant.

We implemented a spatial statistical approach based on an extensive dataset extracted from the French Soil Quality Monitoring Network database^43^. This dataset contains data from over 2200 sites in the France mainland, all of which were analysed for major chemical, physical, pedological, climatic, and geological parameters including Si_CaCl2_ concentrations. We focused on the Si_CaCl2_ soil concentrations of the surface horizons (0–30 cm), considered to be the horizon most explored by plant roots.

## Results and discussion

### Variables explaining Si_CaCl2_ using digital soil mapping (DSM)

Concentrations of Si_CaCl2_ in French topsoils ranged from 2.3 to 134 mg kg^−1^ (*subset 1*, see Supplementary Table S1) with a 1st quartile value of 9.5 mg kg^−1^, a median of 17 mg kg^−1^, and a 3rd quartile of 28 mg kg^−1^ (Table 1). The values fell in the same range as values found elsewhere^25,26^.

**Table 1:** Summary statistics of Si_CaCl2_ concentrations measured for the *subset 1* (Supplementary Table S1).

Soil parent material/type	Land use	Si_CaCl2_ (mg kg^−1^)					n
		Mean	Sd	Q25	Q50	Q75	
Carbonated soils on sediment	All	31	17	20	26^a^	36	516
	Non-cultivated	25	11	17	23	30	219
	Cultivated	35	20	23	29	41	297
Soils on igneous extrusive rock	All	51	27	32	47^b^	66	29
Soils on igneous intrusive rock	All	11	5.7	8.6	10^c^	13	155
	Non-cultivated	12	6.1	8.8	11	14	109
	Cultivated	10	4.2	7.6	9.3	11	46
Soils on metamorphic rock	All	11	5.8	7.8	9.6^c^	13	217
	Non-cultivated	11	5.8	7.5	9.5	13	120
	Cutivated	12	5.9	8.1	9.7	14	97
Non-carbonated soils on sediment	All	20	15	9.2	16^d^	28	1013
	Non-cultivated	16	13	7.9	12	21	537
	Cultivated	25	16	12	23	34	476
Podzols	All	5.1	2.8	3.7	4.2^e^	4.9	56
All data *(subset 1)*		21	16	9.5	17	28	1986

The summary statistics are provided for the combination of a parent material group and land use. Q25, Q50, and Q75 are the 25%, 50%, and 75% percentile, respectively. Medians sharing a letter are not significantly different according to pairwise comparisons of Wilcoxon test with Tukey adjustment for multiple comparisons.

**Figure 1:**
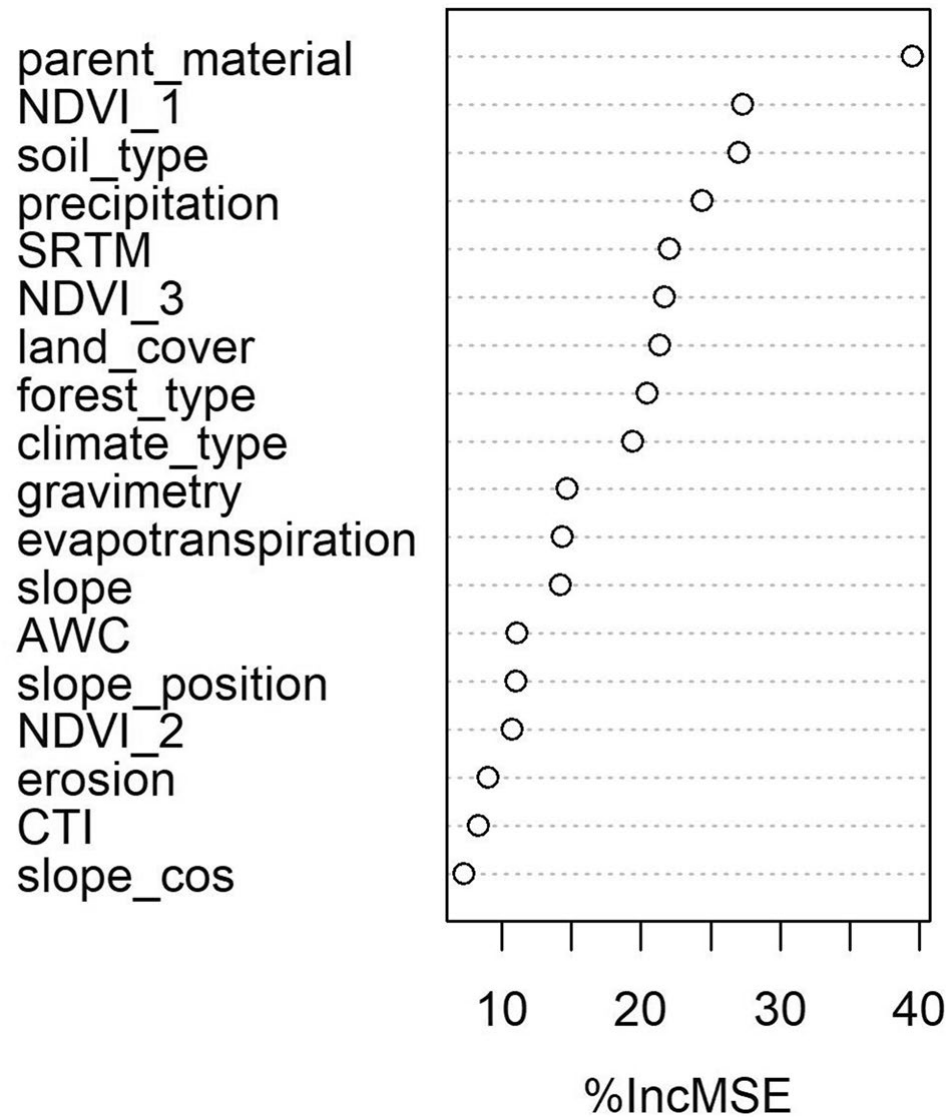
Importance of variables in the Random Forest model (in % of increasing Mean Squared Error, %IncMSE).

**Figure 2:**
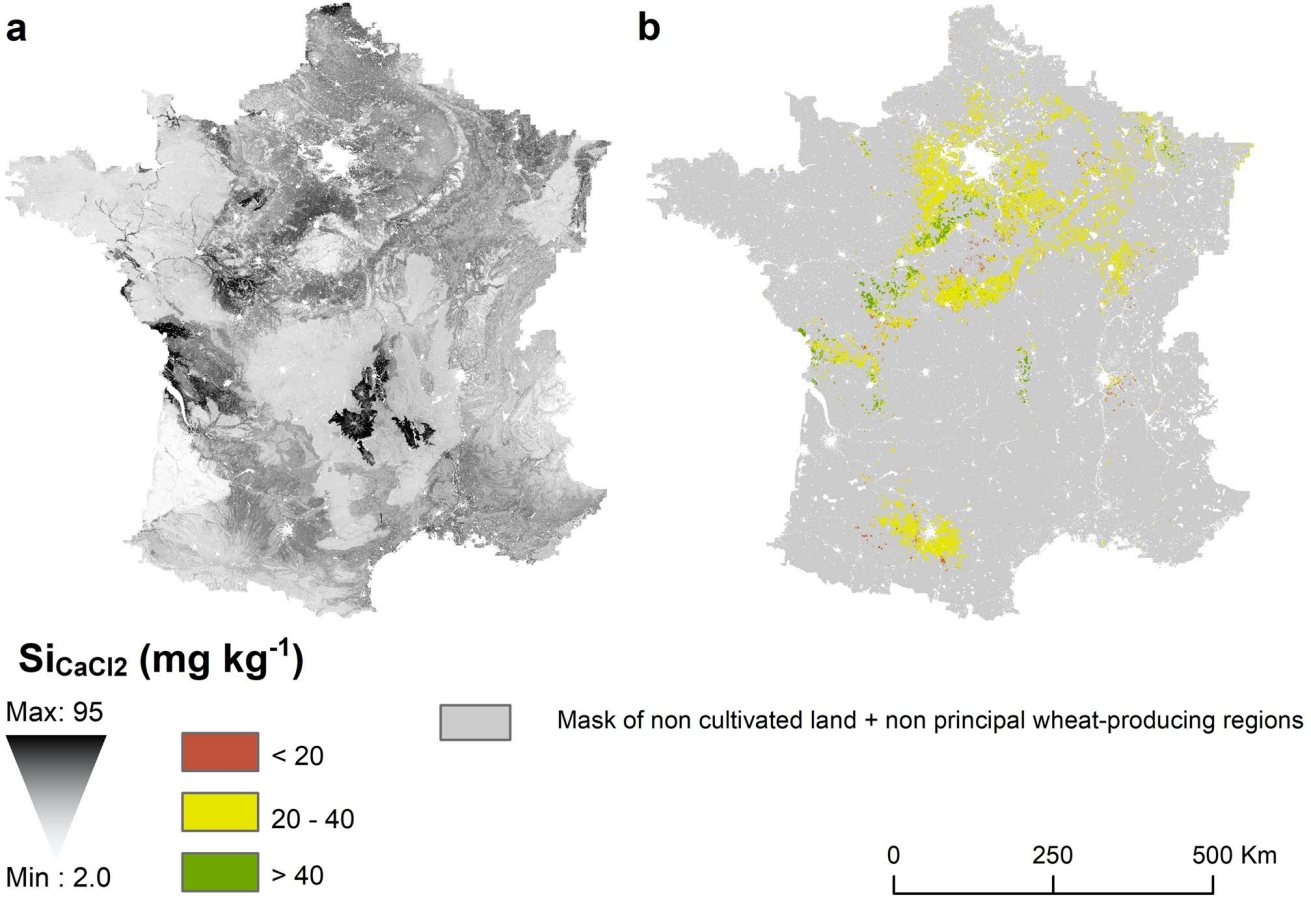
Regression Kriging predictions of Si_CaCl2_ concentrations in French topsoils at 90 m resolution. (**a**) continuous colour gradient representation; (**b**) categorical representation in 3 classes of Si_CaCl2_ concentrations based on critical values defined for sugarcane and rice^28,29,31^ for soils cropped in wheat, obtained by crossing the ‘arable land’ pixels, ‘permanent crop’ and ‘heterogeneous agricultural’ areas with the exception of ‘agro-forestry’ of Corine Land Cover classes^54^ with municipalities for which type of farming is primarily cereal crop, according to French OTEX classification (Orientation Technico-Economique des Exploitations). We removed the municipalities from 7 departments (Ain, Haut-Rhin, Bas-Rhin, Gironde, Pyrénées-Atlantiques, Hautes Pyrénées, Landes) where corn is the main cereal under production. Maps were generated using ArcGIS software version 10.7.1 (ESRI: https://www.esri.com/en-us/home).

The spatial distribution of Si_CaCl2_ at the French territory scale obtained by the DSM approach was evaluated by the following criteria using a 30-fold cross validation process: R^2^ = 0.43, concordance = 0.58, RMSE = 13 mg kg^−1^, and bias = 0.24 mg kg^−1^. These criteria are of the same order of magnitude than those obtained in other DSM studies, at national scale, for other soil parameters, which yield R^2^ ranging from 0.25 to 0.55^44–46^. The most important covariates for predicting the spatial pattern of the Si_CaCl2_ concentration in the obtained Random Forest model, was, as expected, the parent material (*parent_material*) (Fig. 1). The second most important covariate was the so called *NDVI_1,* a proxy for vegetation growth dynamics as defined by Loiseau et al.^47^. Other important covariates for the model were soil type, precipitation, and land use, in that order. These results confirmed the data obtained in Japan^28^ and in Louisiana^31^ indicating that PAS depends on pedological/geological conditions. As a consequence, the primary spatial structures of the map (Fig. 2a) matched the parent materials and the negative correlation with total Si concentration^29^. These results also highlighted the importance of land use/vegetation on the Si_CaCl2_ concentration.

### Physical and chemical control of Si_CaCl2_ in non-cultivated soils

In this part, only non-cultivated soils are considered to study the mechanisms that occur naturally in soils, without the influence of agriculture that generally strongly modifies some of the soil characteristics considered as PAS drivers, notably the soil pH by liming for slightly acidic to acidic soils, the soil organic carbon concentration (SOC).

#### Overall correlations between the main soil characteristics and Si_CaCl2_ concentrations

In the non-cultivated soils, we confirmed previous findings indicating that Si_CaCl2_ concentration was positively correlated (Table 2) with pH (r = 0.46) and the < 2 µm fraction (r = 0.59). The relationships with SOC and iron oxides (estimated by the Mehra Jackson extraction method^48^) were weaker (with r of 0.27 and 0.35, respectively). Si_CaCl2_ was also positively correlated with CEC (r = 0.59), exchangeable Ca (r = 0.54), and CEC of < 2 µm fraction (r = 0.44). The correlation of Si_CaCl2_ with pH indicated that Si_CaCl2_ concentration may be controlled by Si adsorbed on the surface of soil minerals and/or by the soil weathering state^25,26^. The correlation of Si_CaCl2_ with the < 2 µm fraction can be attributed to the presence of small phytoliths^49^, clay mineral dissolution^50^, or adsorption onto clay minerals^51^.

**Table 2:** Correlation coefficients between Si_CaCl2_ and pH, < 2 µm, and CEC of the < 2 µm fraction (< 2 µm CEC) for the non-agricultural soils as a whole and by classes of parent material.

Class	Si_CaCl2_ ~ pH	Si_CaCl2_ ~ (< 2 µm fraction)	Si_CaCl2_ ~ (< 2 µm CEC)	n
All non-cultivated soils	**0.46**	**0.59**	**0.44**	1065
Carbonated soils on sediment	− 0.099	**0.2**	0.093	219
Soils on igneous extrusive rock	**0.63**	**0.66**	**0.63**	27
Soils on igneous intrusive rock	**0.25**	**0.39**	**0.36**	109
Soils on metamorphic rock	**0.3**	**0.42**	**0.3**	120
Non-carbonated soils on sediment	**0.48**	**0.68**	**0.48**	537
Podzols	0.034	**0.81**	0.15	53

Significant correlations (p value < 0.05) are reported in bold.

Medians sharing a letter are not significantly different according to pairwise comparisons of Wilcoxon test with Tukey adjustment for multiple comparisons.

**Figure 3:**
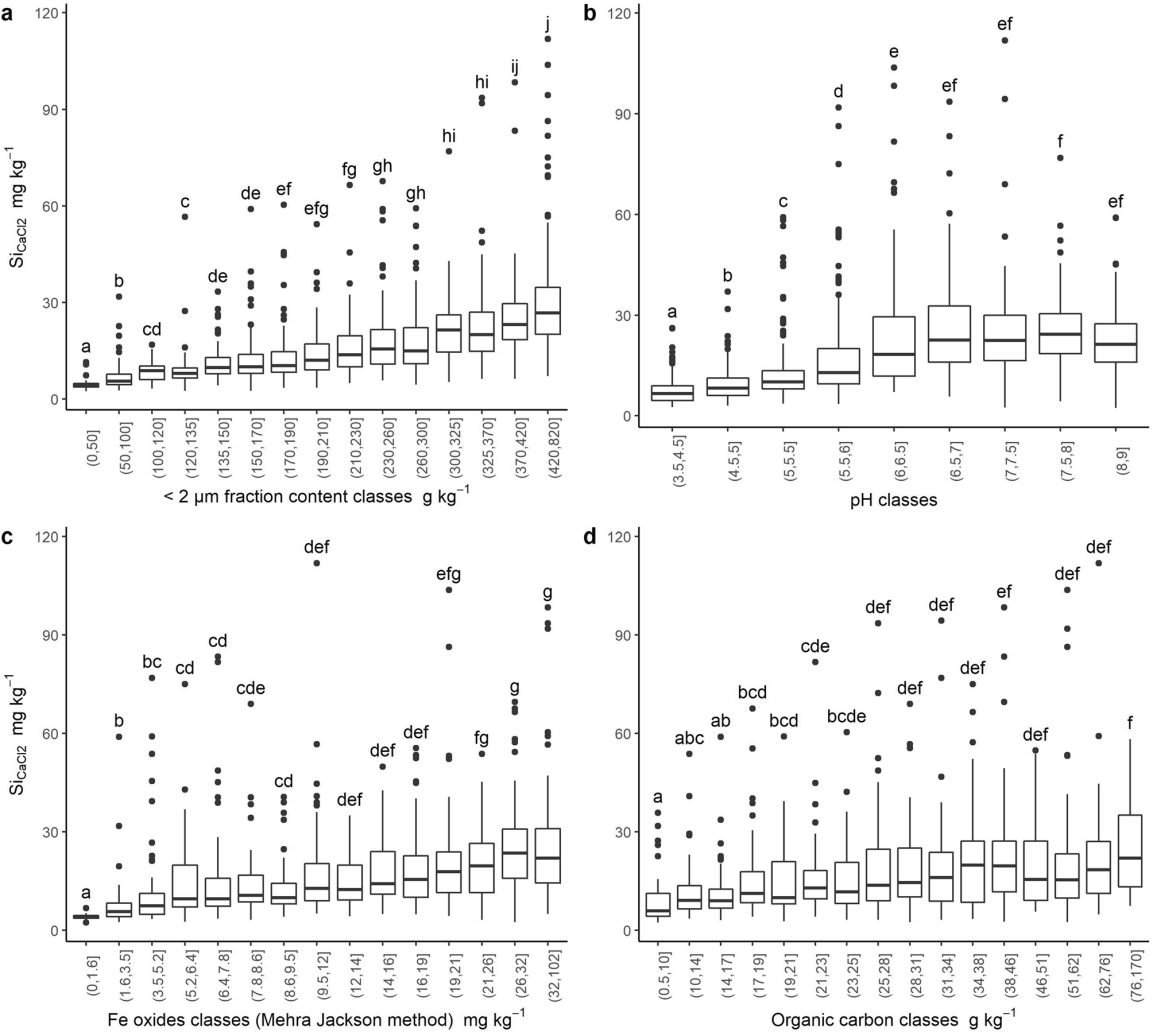
Boxplots of Si_CaCl2_ concentration as a function of (**a**) < 2 µm fraction content classes, (**b**) pH classes , (**c**) Fe oxides concentration (estimated by the Mehra Jackson method) classes (**d**) soil organic carbon content classes for non-cultivated soils only. Classes have been created so that uncertainty around the central value does not overlap with the uncertainty around the central value of the surrounding classes. Classes with less than 40 individuals were merged. Groups of individuals sharing a letter are not significantly different according to pairwise comparisons of Wilcoxon test with Tukey adjustment for multiple comparisons.

**Figure 4:**
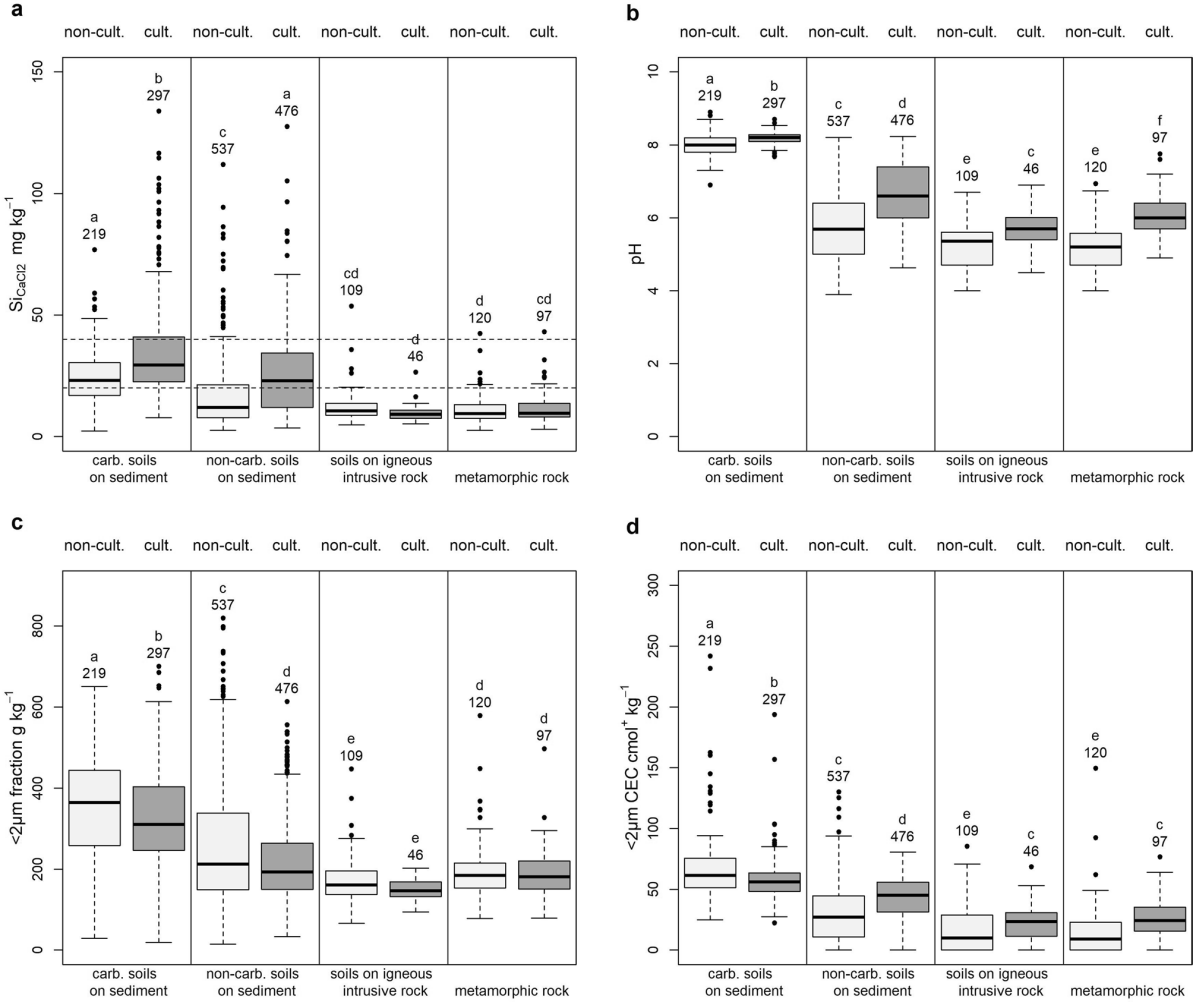
Boxplots by parent materials and land uses of: (**a**) Si_CaCl2_, (**b**) soil pH, (**c**) < 2 µm fraction content, and (**d**) < 2 µm CEC. Groups of individuals sharing a letter are not significantly different according to pairwise comparisons of Wilcoxon test with Tukey adjustment for multiple comparisons.

Nevertheless, while Si_CaCl2_ linearly increased with the < 2 µm fraction in non-cultivated soils regardless of parent material (Fig. 3a), the correlation with pH was more complex. The concentration in Si_CaCl2_ increases with pH for pH values lower than 6–7 and then slightly decreases for pH larger than 8 (Fig. 3b). Such an evolution of Si_CaCl2_ with pH was already described in the literature^52^. As a consequence, there was no correlation between Si_CaCl2_ and the soil pH (Table 2) for carbonated soils due to their high (> 7) and rather constant pH values (Fig. 4b). For the SOC and Fe oxides, the relationship with Si_CaCl2_ is less marked, even though the Si_CaCl2_ concentration globally increases with these parameters (Fig. 3c,d). As a conclusion, in non-cultivated soils, the Si_CaCl2_ concentration seems to be mainly driven by < 2 µm content and pH suggesting adsorption.

#### The impact of pH on the Si_CaCl2_ concentration is a function of the < 2 µm fraction

**Figure 5:**
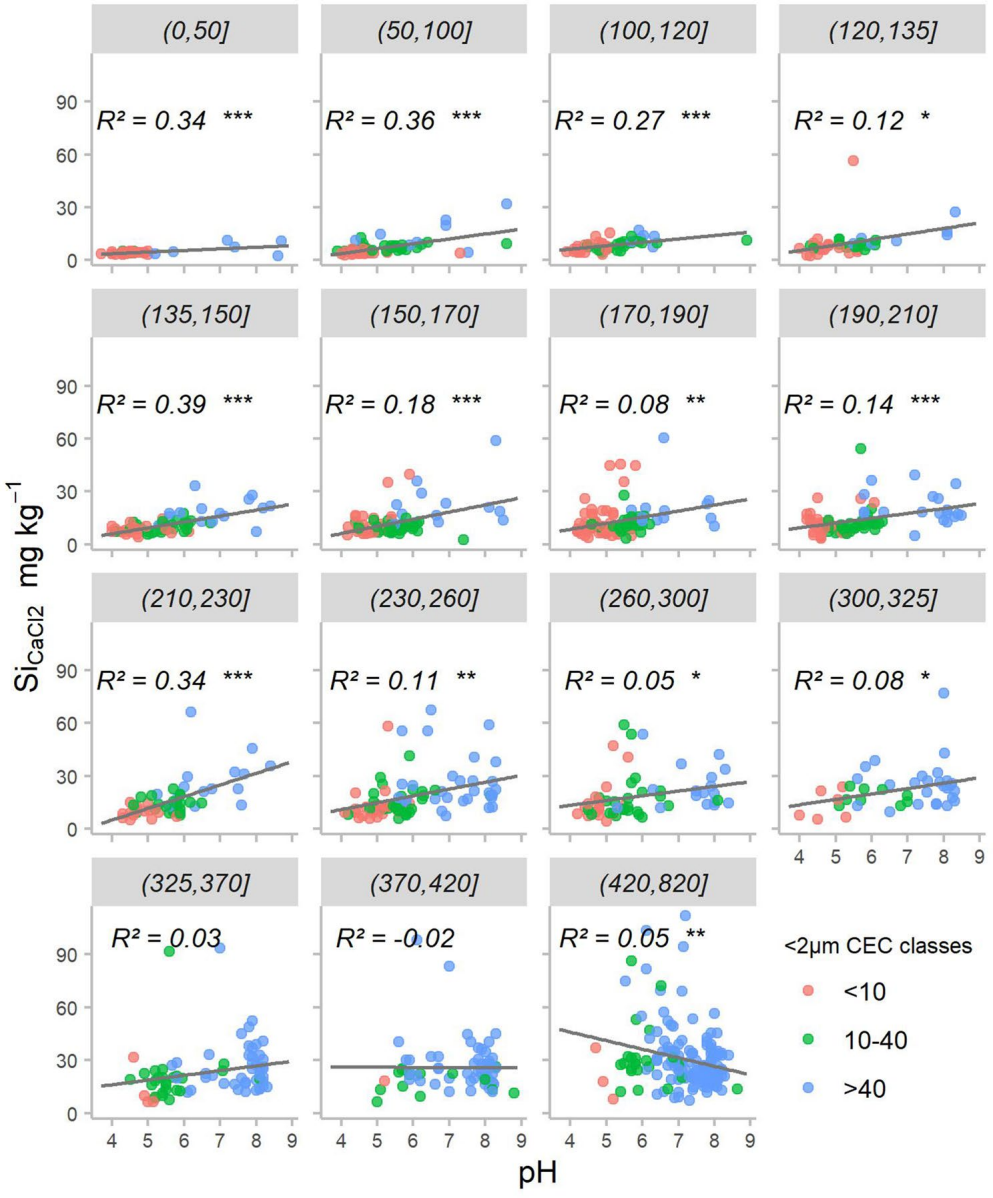
Relationship between topsoil Si_CaCl2_ and pH for the different < 2 µm content classes defined in Fig. 3, for non-cultivated soils. The classes of < 2 µm contents are expressed in g kg^−1^ and reported in the grey box at the top of the corresponding graph. The colours identify the classes of < 2 µm CEC corresponding to the classes of clay mineral CEC values provided by Goldberg et al.^53^. Observations with < 2 µm CEC values larger than 100 cmol^+^ kg^−1^ were eliminated (20 observations). Associated R^2^ values and levels of significance are reported: ≤ 0.001 ‘***’, [0.001;0.01] ‘**’, [0.01, 0.05] ‘*’, > 0.05 ‘ ’.

To explore the respective role of pH, CEC of < 2 µm fraction, and < 2 µm fraction on Si_CaCl2_ concentrations in soils, we considered the correlation between soil Si_CaCl2_ concentration and soil pH for each of the < 2 µm fractions defined in Fig. 3a. For low < 2 µm fraction concentrations (< 50 g kg^−1^), the Si_CaCl2_ concentration was significantly correlated with the pH but the slope of the regression was very low and appeared to be driven by a few extreme values (Fig. 5). As a result, Si_CaCl2_ concentrations increased minimally from 3.3–4.3 mg kg^−1^ to 6.4–9.3 mg kg^−1^ in 4.5 pH units, demonstrating the low impact of pH on Si_CaCl2_ concentration for soils with poor < 2 µm fractions. For < 2 µm fraction concentrations ranging from 50 to 325 g kg^−1^, Si_CaCl2_ concentration increased with pH. Nevertheless, for < 2 µm fraction content higher than 230 g kg^−1^, the increase of Si_CaCl2_ with pH is smaller. No correlation is observed for < 2 µm fraction concentrations higher than 325 g kg^−1^, indicating that adsorption might not be the dominant process at these < 2 µm contents. Carbonated soils are in this < 2 µm fraction range (median of 364 g kg^−1^, Supplementary Table S2) explaining the absence of correlation of Si_CaCl2_ with pH.

As a conclusion, the classically described increase of Si_CaCl2_ with pH (explained by Si adsorption processes) was only encountered in temperate soils for non-carbonated soils with < 2 µm concentrations higher than 50 g kg^−1^ or lower than 325 g kg^−1^.

#### Impact of the nature of the < 2 µm fraction on Si_CaCl2_ concentration

The < 2 µm CEC can be used as a proxy for the nature of the clay minerals of the soil as shown by the contrasted CEC of the different clay minerals provided by Goldberg et al.^53^. On this basis^53^, we assumed that (i) soils with < 2 µm CEC lower than 10 cmol^+^ kg^−1^ were primarily kaolinitic or possessed a < 2 µm fraction rich in quartz; (ii) soils with < 2 µm CEC ranging from 10 to 40 cmol^+^ kg^−1^ contained a large amount of illite and chlorite or a complex mixture of clay minerals; and (iii) soils with a < 2 µm CEC larger than 40 cmol^+^ kg^−1^ contained a large quantity of vermiculite and smectite.

A correlation of the Si_CaCl2_ concentrations with the < 2 µm CEC (a proxy for the nature of the clay minerals of the soil) was observed globally and for all non-carbonated parent material, with the exception of podzols (Table 2), suggesting a role of the nature of the clay minerals on the Si_CaCl2_ concentrations. However, Fig. 5 shows that soil pH and clay nature were linked, with acidic soils being mainly kaolinitic and basic soils being more smectitic or rich in vermiculite. This could partially explain the link between Si_CaCl2_ concentration and pH, because kaolinites are more stable than other clay minerals (e.g. smectite)^14^.

For carbonated soils developed on sedimentary parent materials and podzols, the Si_CaCl2_ concentrations did not appear to be impacted by the nature of the corresponding clay mineral (< 2 µm CEC) (Table 2). In carbonated soils on sediment parent material, the clay mineral assemblage was primarily dominated by smectite and vermiculite, according to the < 2 µm CEC recorded (Fig. 4d). In podzols, the nature of the clay minerals did not vary much and primarily consisted of kaolinite and quartz in the < 2 µm fraction (see the very low < 2 µm CEC in Supplementary Table S2).

Our results showed that the increase in Si_CaCl2_ concentrations in tandem with pH may be explained by changes in the nature of the underlying mineralogy of the < 2 µm fraction. In acidic soils with kaolinite as the primary clay component, the Si_CaCl2_ was lower than in soils with a near neutral pH, with a primary clay component of smectite.

### Impact of agriculture on available silicon in soils

For soils derived from carbonated and non-carbonated sediment material (73% of French soils), Si_CaCl2_ significantly increased in the cultivated soils (perennial and annual crop) as compared to soils at uncultivated sites (Fig. 4a). Indeed, the results of linear model showed that about 12% of the total variance is explained by this difference. However, for soils derived from igneous intrusive rock and metamorphic rock, there was no significant difference in Si_CaCl2_ concentrations between cultivated and non-cultivated sites. For these two parent materials, Si_CaCl2_ concentrations were very low, as compared to other parent materials. Thus contrarily to what was expected, cultivation increases Si_CaCl2_ concentrations but only in soils where this concentration is not too low.

Cultivated soils exhibited significantly higher pH values than uncultivated vegetated soils, regardless of parent material (Fig. 4b) due to liming practices on cultivation, with the notable exception of the carbonated soils, which are generally not limed. For carbonated soils, the difference of pH was due to acidifying conditions under non-cultivation, as compared to agricultural conditions. The difference in pH between cultivated and non-cultivated soils was less than 0.5 for carbonated soils on sediment and igneous intrusive rocks. Since carbonated soils have a < 2 µm fraction content higher than 325 g kg^−1^, pH has no effect on the Si_CaCl2_ concentration as shown by Fig. 5 and Table 2.

For non-carbonated soils on sediments and soils on metamorphic rocks, the difference was higher: the average pH of non-cultivated soils was 5.7 and 5.2, while the average pH of cultivated soils was 6.7 and 6.1 respectively for non-carbonated soils on sediments and metamorphic rocks (Supplementary Table S2).

As shown in Fig. 4c (and Supplementary Table S2), the < 2 µm fractions for non-carbonated soils on sediments primarily comprised 50 to 325 g kg^−1^. For this < 2 µm fraction range, we showed that the Si_CaCl2_ concentration increased with pH (Fig. 5). Therefore, cultivation associated to a pH increase by liming may be responsible for the Si_CaCl2_ concentration increase under cultivation for these soils. This pH increase was also associated with an increase in < 2 µm CEC and therefore with a higher smectite/vermiculite content under cultivation, and a higher illite/chorite content under permanent vegetation (Fig. 4d). Similar changes in clay mineral composition following changes in land use were observed on paired site approaches (Cornu et al.^54^ and references herein). The pH-driven effects of liming on Si_CaCl2_ concentrations have already been observed^52^. Indeed, increases in pH could cause higher adsorption of silicon to soil minerals and facilitate the solubilization of phytoliths in the soil solution, thus increasing the Si_CaCl2_ concentration at short time scales^52^.

### Consequences in terms of potential PAS deficiency for wheat in temperate soils

To our knowledge, no study has defined the critical level of Si_CaCl2_ for avoiding Si deficiency for temperate staple crops as wheat. Critical levels are, however, available in the literature for rice and sugarcane; these levels are adopted as references for discussion here because wheat has a shoot Si concentration between those of rice and sugarcane^10^. We proposed two critical values: a lower bound (20 mg kg^−1^) defined by Haysom and Chapman^33^ for sugarcane in Australia, and an upper bound (40 mg kg^−1^), an average value provided for rice based on data from silt loam in Louisiana soils^31^ (37 and 43 mg kg^−1^) and from southern acidic soils in India^30^ (43 mg kg^−1^). We applied these two thresholds to the French soils cultivated with wheat, obtained by crossing the arable land pixels defined to Corine Land Cover map^55^ with the cereal producing municipalities from the French OTEX classification (the Technico-economic orientation of the farms, http://agreste.agriculture.gouv.fr), to quantify the importance of potential Si_CaCl2_ deficiency in temperate soils (Fig. 2b). It results that only 4% of the soils cultivated with wheat fell below the 20 mg kg^−1^ critical level and could therefore be depleted of plant available Si. However, this hypothesis requires a more technical definition of the Si requirements of wheat. Indeed, this would enable to determine if the soils having concentrations between 20 and 40 mg kg^−1^ and representing 85% of the surfaces cultivated with wheat are below or under the critical level for wheat.

## Materials and methods

### Soil data from RMQS program

The soil data used in this study were provided by the French Soil Quality Monitoring Network (RMQS). The RMQS network consists of observation sites situated at the centre of a regular grid (16 * 16 km) covering the French territory. It provides 2111 sites in metropolitan France, almost half of which are cultivated (permanent crops or field crops) and the remaining of which consist of pastures, natural vegetation, or urban soils. The dataset used in this study corresponds to the first sampling campaign of the RMQS carried out from 2000 to 2009.

Soil type, parent material, climate, and land use were described in the field. At each site, 25 core samples were taken within a 20 m × 20 m plot and combined into a composite sample. Sampling depth generally consisted of the 0–30 cm layer^43^. The composite samples were air-dried and sieved to 2 mm before being analysed in Soil Analysis Laboratory of INRAE (Arras, France).

The following parameters were measured: (i) the total soil organic carbon concentration, as measured by dry combustion (NF ISO 10694); (ii) the particle size distribution, by wet sieving and pipette method (NF X 31-107); (iii) cation exchange capacity (CEC) and exchangeable cations (cobaltihexamin method, NF X 31-130); (iv) pH in water (1 to 5 soil to water ratio, NF X 31-107); (v) calcium carbonate, using the volumetric method (NF X 31-106) (CaCO_3_); and (vi) total P, K, Ca, Mg, Fe, and Al, determined by ICP-MS after dissolution with hydrofluoric and perchloric acids (NF X 31-147). The < 2 µm CEC was estimated as follows:

1 $$2\,\upmu\text{m}\;\;CEC=\frac{\left(CEC-0.15*OC\right)}{<2\,\upmu \text{m}\;fraction\; content}*1000$$

with CEC in cmol^+^ kg^−1^, OC in g kg^−1^, and < 2 µm faction content in g kg^−1^. We used 0.15 cmol^+^ kg^−1^ as an estimate of the CEC of organic matter.

Total Si was analysed from a subset of 673 samples by ICP after alkaline fusion^56^, and extrapolated to the remaining sites using the following conceptual equation:

2 $${\text{Si}} = f\left( {{\text{Al}},\;{\text{Fe}},\;{\text{K}},\;{\text{Na}},\;{\text{Ca}}_{nc} ,\;{\text{Mg}}_{nc} ,\;{\text{P}},\;SOC,\;{\text{CaCo}}_{3} ,\;residual\;water} \right)$$

where *Ca*_*nc*_ and *Mg*_*nc*_ are the fractions of Ca and Mg, respectively, that are not included in carbonate minerals or adsorbed to the exchangeable surfaces, and SOC is the organic carbon percentage. This model was implemented in cubist^29^ and yielded an R^2^ value greater than 0.98.

Bioavailable Si (Si_CaCl2_) was estimated on samples from 2091 sites using the 0.01 M CaCl_2_ method^33^. This widely adopted method^25^ allows estimation of the pool of readily soluble Si.

Bulk samples were equilibrated during 16 h at room temperature with 0.01 M CaCl_2_ with a solid:liquid ratio of 1:10. Si was then analyzed, after filtration of the supernatant at 0.45 µm, by Inductively Coupled Plasma Atomic Emission Spectroscopy (axial ICP-AES; 720 ES, Varian)^29^. The limit of quantification of the method was of 0.5 mg kg^−1^ with an uncertainty, U, (with a confidence level of 0.95) evaluated as follow:

3 $$U=0.0271*{\mathrm{Si}}_{\mathrm{CaCl}2}+0.25$$

with Si_CaCl2_ and U expressed in mg kg^−1^.

To decipher the impact of agricultural use on Si_CaCl2_ concentration, we stratified the database by (i) parent material, based on the European Soil Information System (EUSIS) classification^57^ available at the French territory scale as in Landré et al.^29^; and by (ii) land use. The group of soils developed on sediment was cut into two subgroups (carbonated and non-carbonated depending on their carbonate content (> 1% and < 1% respectively). We considered soils under forests, pastures, parks, natural vegetation, and wetlands to be non-cultivated soils. We did not make any distinction of land use for soils on igneous extrusive rocks (volcanic) and podzols, because cultivation of these soil types was limited to two and three sites, respectively.

Our statistical analysis was based on a subset of 1986 data points (*subset 1,* see Supplementary Table S1) due to the removal from the initial 2091 point dataset of any sites with missing geological information and three sites with peat soils.

### Digital soil mapping approach

To map and define the spatial distribution of Si_CaCl2_ concentrations, we implemented a digital soil mapping (DSM) approach based on the *scorpan* model framework proposed by McBratney et al.^58^, a spatial prediction function utilizing quantitative relationships between soil properties and soil forming factors as follows:

4 $${Soil}_{x}=f\left(s,c,o,r,p,a,n\right)+e$$

where $Soil$ is a soil property at position $x$. The $s$ refers to soil information derived from prior soil maps or from remote or proximal sensing data; $c$ refers to the climatic properties of the environment at a given point; $o$ refers to organisms, including vegetation, fauna, or human activity; $r$ refers to relief; $p$ refers to the parent material or lithology; $a$ refers to the soil age; $n$ refers to space or spatial position; and $e$ is the spatially correlated errors.

To apply this model, we retained a set of spatial covariates describing *scorpan* factors to predict the spatial distribution of Si_CaCl2_ at 90 m resolution. All covariates were previously resampled at the same resolution. The selection was carried out from a larger set of covariates using a combination of expert knowledge and statistical approaches. The latter was based on the *boruta* package in R^59^. This algorithm uses the classification of the covariate importance implemented in the *randomForest* package and compares the importance with random variables based on the Z score value. This step allows the identification of the most relevant covariates before fitting a prediction model. The initial 18 spatial covariates represented the *scorpan* factors as follows: *soil* (map of soil type and available water capacity (AWC)), *climate* (map of climate type, precipitation, and evapotranspiration), *vegetation* (land use, forest type), *parent material* (parent material types and gravimetry), and *relief* (shuttle radar topography mission (SRTM), compound topographic index (CTI), slope cosine, erosion, slope, and slope position). A final covariate corresponding to the normalized difference vegetation index (NDVI) was derived from remote sensing data. This spectral index is widely used to describe the photosynthetic capacity of vegetative cover. The underlying assumption driving this method holds that changes in vegetation may reflect various plant responses to climate, land management, or soil properties^47^. This covariate was computed from a large time series dataset. To summarise the temporal data, Loiseau et al.^47^ performed a principal component analysis that yielded 3 covariates corresponding to the 3 first components: NDVI_1, NDVI_2, and NDVI_3.

For the *scorpan* model, we developed a regression kriging (RK) model using the selected covariates and soil observations. The RK model is a hybrid technique combining a Random Forest approach with a geostatistical approach. Random Forest^60^ consists of an ensemble of regression trees built from covariates and point data. The final prediction is the mean of the individual tree predictions. This technique is applicable in R and requires two main parameters: the number of randomly selected splitting variables at each tree (mtry), and the number of trees (ntree). We used the default parameters provided by the package (mtry = 6 and ntree = 500). The final predictions are the sum of the Random Forest predictions and the residuals computed through an ordinary kriging procedure^61^.

The RK model was fitted on the on a subset of the RMQS sites for which covariate information were available (*subset 2:* 1987 points, see Supplementary Table S1). This procedure was implemented with the package *GSIF*^62^, which also allows fitting of residuals in a variogram.

To assess the covariates importance in the model, we used the function varImp() implemented in the *Random Forest* package. The function calculates the difference in MSE (Mean Squared Error) when the values of each predictor are shuffled. This indicator is then normalized by the standard deviation of the differences to obtain the Increase in MSE noted %IncMSE. The highest values are attributed to the more important variables.

The validity of the fitted prediction model was evaluated by a 30-fold cross validation. At each step, the soil dataset was split into two datasets: 2/3 for calibration, and 1/3 for validation.

To obtain an estimate of the final map accuracy, the validation indicators were calculated at each iteration and then averaged over the 30 repetitions. We used different indicators for validation, including the Root Mean Square Error (RMSE), the coefficient of determination (R^2^), the concordance^63^, and the bias.

### Take home messages

The Si_CaCl2_ concentrations in French soils is highly variable (from 2.3 to 134 mg kg^−1^) and depends mainly on parent material and soil type, but also on the land use.

When the soils are cultivated, the concentration in Si_CaCl2_ significantly increases for soils developed on sediment parent material but not for those developed on metamorphic rocks and igneous intrusive rocks. For these two last parent materials, Si_CaCl2_ concentration is low compared to soils developed on sediments. For soil developed on igneous extrusive rocks and podzol, no conclusion on the impact of agriculture on Si_CaCl2_ concentrations could be drawn due to the low number of sites under cultivation for these soils in France. For non-carbonated soils, this increase is due to the pH increase associated to liming practices. More research is needed to better understand the impact of cultivation on Si_CaCl2_ concentration for carbonated soils for example through a paired sites approach.

We also verified on this large panel of temperate soils that the Si_CaCl2_ concentration is governed by the < 2 µm fraction, pH, and iron oxide content to a lesser extent. The nature of the clay assemblage seemed also to act on the Si_CaCl2_ concentration. We however showed that the Si_CaCl2_ concentration increases with pH only for soils with content in < 2 µm fraction ranging from 50 to 325 g kg^−1^.

We also showed that 4% of the soils cropped with wheat could be deficient in Si_CaCl2_. This result was based on critical level of Si_CaCl2_ estimated for sugar cane and rice. Studies should be done to determine precisely the critical level for wheat.

At last, this study did not allow accessing the kinetic aspects. Other Si pools and/or chronosequences studies should be analyzed to answer this question.

## Supplementary information

Supplementary Information.

## Data Availability

The dataset analysed during the current study can be retrieved from https://doi.org/10.15454/CFWBAA^64^. However, the location information is not publicly accessible because the data contain confidential information.
