# Supplementary material for: Agriculture increases the bioavailability of silicon, a beneficial element for crop, in temperate soils
Source: Sci Rep. 2020 Nov 17;10:19999. doi: 10.1038/s41598-020-77059-1 (PMC7672074; doi:10.1038/s41598-020-77059-1)
Supplement: Supplementary file 1 — Supplementary Information. [file 41598_2020_77059_MOESM1_ESM.pdf]

Agriculture increases the bioavailability of silicon, a beneficial element for crop, in temperate soils

M. Caubet[a], S. Cornu[b*], N.P.A. Saby[a], J-D. Meunier[b]

a) INRAE, Infosol, US 1106, Orléans, France

b) Aix-Marseille Univ, CNRS, IRD, Coll de France, INRAE, CEREGE, Aix-en-Provence, France

***Corresponding author:** S. Cornu sophie.cornu@inrae.fr

Supplementary materials

Table S1: Description of the datasets used in the study

	Number of points	Use in the study
Dataset with Si_{CaCl2} information	2091	Not used as such
Sub-dataset with complete information on soil types and parent material, 3 histosols are excluded *(subset 1)*	1986	Data analysis
Sub-dataset with Si_{CaCl2} information and spatial information from covariates *(subset 2)*	1987	Spatial analysis

<u>Table S2:</u> Summary statistics of a set of soil properties measured on the monitoring sites: pH values, < 2 µm fraction concentration and the CEC values of the < 2 µm fraction. The summary statistics are provided for the combination of a parent material group and a land use. Q25, Q50 and Q75 are the 25 %, 50% and 75 % percentile respectively

Soil parent material/type	Land use	pH					<2µm fraction (g kg^{-1})					<2µm fraction CEC ($cmol^{+}$ kg^{-1})					n
		mean	sd	Q25	Q50	Q75	mean	sd	Q25	Q50	Q75	mean	sd	Q25	Q50	Q75	
**carbonated soils on sediment**	all	8.1	0.24	8.0	8.1	8.2	339	125	252	329	423	61	22	50	58	67	516
	non-cultivated	8.0	0.28	7.8	8.0	8.2	357	129	258	364	444	67	27	51	61	75	219
	cultivated	8.2	0.17	8.1	8.2	8.3	326	120	246	310	403	57	16	48	56	63	297
**soils on igneous extrusive rock**	all	5.7	0.63	5.3	5.7	6.0	256	102	188	241	278	23	27	1.0E-04	12	52	29
**soils on igneous intrusive rock**	all	5.4	0.60	4.9	5.4	5.8	164	50	134	152	186	19	18	1.8	15	31	155
	non-cultivated	5.2	0.59	4.7	5.4	5.6	171	56	137	161	195	17	19	1.0E-04	9.9	29	109
	cultivated	5.7	0.47	5.4	5.7	6.0	149	27	132	146	168	23	14	11	23	31	46
**soils on metamorphic rock**	all	5.6	0.74	5.0	5.6	6.1	191	64	152	183	217	20	19	4.6	17	31	217
	non-cultivated	5.2	0.63	4.7	5.2	5.6	193	69	154	184	214	15	21	1.0E-04	9.0	23	120
	cutivated	6.1	0.58	5.7	6.0	6.4	188	56	151	181	220	25	14	16	24	35	97
**non-carbonated soils on sediment**	all	6.2	1.0	5.4	6.1	7.0	237	130	149	203	299	36	21	20	37	51	1013
	non-cultivated	5.7	0.95	5.0	5.7	6.4	255	148	149	212	338	30	23	11	27	45	537
	cultivated	6.7	0.85	6.0	6.6	7.4	218	102	150	193	264	43	16	31	45	56	476
**podzols**	all	4.5	0.40	4.3	4.5	4.6	57	62	22	30	55	4.3	8.6	1.0E-04	1.0E-04	3.7	56
